# Change in the responsiveness of interferon-stimulated genes during early pregnancy in cows with Borna virus-1 infection

**DOI:** 10.1186/s12917-016-0883-5

**Published:** 2016-11-14

**Authors:** Tadashi Takino, Taku Okamura, Tatsuya Ando, Katsuro Hagiwara

**Affiliations:** 1 School of Veterinary Medicine, Rakuno Gakuen University, Ebetsu, Hokkaido Japan; 2 Scientific Feed Laboratory Co., Ltd., 3-5 Miyahara, Takasaki, Gunma Japan; 3 Veterinary Clinical Center, NOSAI Hokkaido, Ishikari district, Japan

**Keywords:** Interferon-stimulated gene, IFN-τ, pregnant, Borna disease virus

## Abstract

**Background:**

Borna disease virus is a neurotropic pathogen and infects the central nervous system. This virus infected a variety of animal species including cows. The most of cows infected with Borna disease virus 1 (BoDV-1) exhibit subclinical infection without any neurological symptoms throughout their lifetime. We previously reported on the low conception rates in-seropositive cows. Interferon-τ (IFN-τ) plays an important role in stable fertilization, and is produced from the fetal side following embryo growth at 15–40 days of pregnancy. IFN-τ induces the expression of interferon-stimulated gene (ISG) 15 and Mx2 in peripheral blood mononuclear cells (PBMCs). To understand the embryo growth and maternal reaction during early pregnancy in cows with BoDV-1 infection, we aimed to assess the gene expression of ISG15 and Mx2 from PBMCs in BoDV-1-seropositive cows.

**Results:**

None of the cows showed any clinical and neurological symptoms. Among the cows that conceived, the expressions of the ISG15 and Mx2 genes were greater in the BoDV-1-seropositive cows than in the BoDV-1-seronegative cows; the difference was significant between the cows that conceived and those that did not (*P* < 0.05).

**Conclusions:**

The expression of ISG15 and Mx2 genes during early pregnancy significantly increased in the BoDV-1-seropositive cows and may be important for the maintenance of stable pregnancy in BoDV-1-infected cows. In contrast, the gene expression levels of ISG15 and Mx2 did not significantly increase during early pregnancy in BoDV-1-seronegative cows. Thus, BoDV-1 infection may lead to instability in the maintenance of early pregnancy by interfering with INF-τ production.

## Background

Borna disease virus is a negative single-stranded RNA virus that was first identified in Germany [1]. The potential hosts for the virus widely range from birds to non-human primates, such as cattle, sheep, rodent and horse [1–4]. The BoDV-1 is a neurotropic pathogen that infects the central nervous system. Several cases of borna disease virus 1(BoDV-1) infection have been reported in Japanese cows, most of which exhibited subclinical infection without any neurological symptoms. Moreover, we previously reported a decrease in the conception rate in BoDV-1 antibody-positive dairy cows. Although ovulation and estrus at the time of artificial insemination (AI) were normal, BoDV-1 antibody-positive cows required repeated insemination and showed a significant increase in the calving-to-conception interval [5].

Interferon (IFN)-τ is a type 1 IFN produced by the conceptus from the fetal side at 15–40 days of pregnancy. It plays an important role in fertilization; in fact, pregnancy can be maintained by suppressing the production of luteolytic pulses of Prostaglandin (PG)-F2α by the endometrium to maintain corpora lutea and their production of P4, the unequivocal hormone of pregnancy [6–8]. IFN-τ doesn’t stimulate interferon stimulated gene (ISG), such as ISG15 and Mx2 in maternal Luminal epithelium and superficial glands. IFN-τ down regulated oxytocin receptor through IRF-2. In pregnant ovine, exsomes stimulate trophecderm cells to proliferate and secrete IFN-τ with regulation of TLR- mediated cell signaling [9].On the other hands, IFN-τ stimulated ISG through STAT1, STAT2 and IRF9 in immune cells like a PBMCs [8]. IFN-τ induce ISG15, Mx1, Mx2, and OAS1 in PBMCs [10–13]. The production of IFN-τ is low limited and is rapidly eliminated from the mother, it is difficult to directly measure the IFN-τ levels from the serum samples of pregnant mothers. Hence, IFN-τ production can be indirectly estimated by measuring the expression of interferon-stimulated genes (ISG) or Mx levels from maternal mononuclear cells [14]. Thus, the levels of ISG15 and Mx2 could serve as an indicator of the maternal conception status during early pregnancy [8, 13, 15]. Moreover, this type 1 IFN has anti-viral activity [8, 16–18]. On the contrary, BoDV-1 inhibits type 1 interferon induction through interferon regulatory factor (IRF) 3 and 7 pathway [6]. In the present study, to understand the embryo growth and maternal reaction to IFN-τ during early pregnancy in cows infected with BoDV-1, we examined the gene expression of ISG15 and Mx2 from PBMCs in BoDV-1 infected cows.

## Methods

### Sample design

We examined a total of 58 multiparous Holstein Friesian cows. All the cows were confirmed to be clinically healthy during the experimental period and did not have a history of treatment for reproductive difficulties prior to the experiment. The examined cows were similarly managed in the farm and received the same programmed feeding. AI was conducted in dried multiparous cows during standing estrus, and the same practitioner performed ovarian inspection via rectal examination. The blood samples were collected from the cows two times before and after AI at approximately 18–25 days; the PBMC fraction was then separated using the Ficoll-Conray centrifugation method (density, 1.086). Early pregnancy status was confirmed in all the cows via a rectal ultrasound scan of the uterus at 30–45 days after AI. The study was covered by approval of the Ethics Committee of the School of Veterinary Medicine, Rakuno Gakuen University in Japan.

### Quantitative RT-PCR

RNA was extracted from the PBMCs and cDNA was synthesized using anchored-oligo (dT) 18 primer (Transcriptor First Strand cDNA Synthesis Kit, Roche). The expression of ISG15 and Mx2 genes was examined using quantitative RT-PCR (LightCycler® FastStart DNA Master SYBR Green I, Roche); the gene expression was normalized to GAPDH [19]. The primer pairs for the detection of the genes are as follows—ISG15 (NM_174366) forward: GGTATCCGAGCTGAAGCAGTT, reverse: ACCTCCCTGCTGTCAAGGT (131–217, 86 bp); Mx2 (NM_173941) forward: CTTCAGAGACGCCTCAGTCG, reverse: TGAAGCAGCCAGCAATAGTG (2071–2302, 231 bp); and GAPDH (XR_082746) forward: GATTGTCAGCAATGCCTCCT, reverse: GGTCATAAGTCCCTCCACGA (546–638, 92 bp).

### Detection of BoDV-1 antibody

Anti-BoDV-1 antibodies were detected using western blotting, with the recombinant BoDV-1 nucleoprotein antigen [20, 21]. Serum samples were diluted at 1:100 with phosphate-buffered saline containing 10% Block Ace (Dainippon Pharmaceutical Co., Osaka, Japan) and 0.05% Tween 20, and western blotting assays were performed. Antibody-antigen complexes were identified using peroxidase-conjugated goat affinity-purified anti-bovine IgG. The obtained antibody findings were compared with the change in the gene expression of the ISG15 and Mx2.

### Statistical analysis

The statistical analysis of BoDV-1 infected cows between conception and conception failure determined by using Chi-square test. Significant differences in the expressions of the ISG15 and Mx2 genes were assayed using one-way analysis of variance. The ISG15 and Mx2 gene expression ratio calculated after AI value/before AI value. The change in the ratio of gene expression between cows that conceived and cows with conception failure was determined by using Student’s t-test.

## Results

The BoDV-1 antibody examination indicated that 38 of 58 cows were seropositive, as shown in Table 1. The same practitioner conducted AI on dried multiparous cows during standing estrus as well as ovarian inspection via rectal examination; all the cows showed a clear estrus for ovulation without any clinical problems.

**Table 1:** Prevalence of Borna disease virus-1 infection and conception status in the study group

Antibody	Conception	Conception failure	Sum
Positive	15	23	38
Negative	11	9	20
Total	26	32	58

The data represent the number of cows. *P* = 0.258, by Chi-square test

A total of 20 cows exhibited seronegative negative results; of these cows, 11 were pregnant after AI and nine had conception failure (conception rate, 55%). In contrast, the conception rate was 39.5% in seropositive cows, including 15 cows that conceived and 23 cow that had conception failure. There were no significantly deference conception between BoDV-1 infected cows and uninfected cows. All the cows were clinically normal and none showed any clinical symptoms, such as those accompanying BoDV-1 infection cases, including neurological symptoms. Blood examination data indicated normal parameters in all the cows during the observation period.

**Fig. 1:**
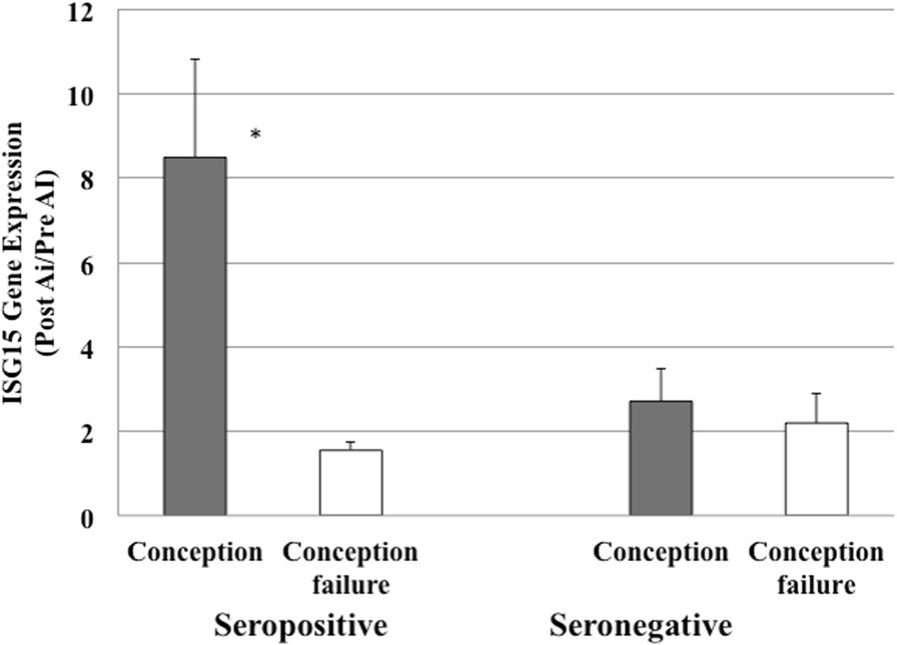
The ratio of ISG 15 gene expression. The value is estimated as the ratio of ISG15 gene expression after AI/before AI, with normalization to GAPDH. * *P* < 0.05. AI, artificial insemination

**Fig. 2:**
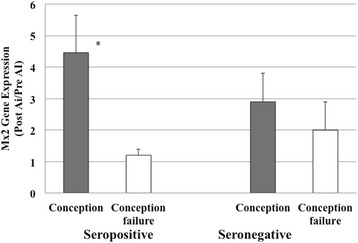
The ratio of Mx2 gene expression. The value is estimated as the ratio of Mx2 gene expressionafter AI/before AI, with normalization to GAPDH. * *P* < 0.05. AI, artificial insemination

The ratio of gene expression was evaluated between two blood-sampling points: before AI and after 18-25 days AI treatment. Among the seropositive cows, the gene expression rate of ISG15 was 8.5 times significantly higher in the cows that conceived as compared to the cows with conception failure (Fig. 1, *p* = 0.0008). The gene expression of Mx2 was also significantly greater (4.5 times) in the conceived cows (Fig. 2, *p* = 0.002). In contrast, among the seronegative cows, the ISG15 and Mx2 gene expression levels in the cows that conceived were only slightly greater than those in the cows with conception failure (Figs. 1 and 2). The gene expression levels of ISG15 and Mx2 after AI were higher in the seropositive cows that conceived than in the cows with conception failure; these levels were significantly different between the cows that conceived and those that did not conceive (*P* < 0.05).

## Discussion

ISG15 is produced from the mononuclear cells in the mother in response to stimulation by IFN-τ. The IFN-τ has also been observed to influence Mx2 gene expression, and the expressions of both ISG15 and Mx2 have been reported during the early stages of pregnancy [13, 15, 22]. In the BoDV-1 infected group, a significant increase in ISG15 and Mx2 expression levels was observed after conception; however, the gene expression levels only slightly increased in the BoDV-1 uninfected group. The cows infected with BoDV-1 tended to increase IFN-τ production and/or a higher response to IFN-τ during pregnancy. The secretion of IFN-τ is regulated by exsome, endogenous jaagsiekte retroviruses in ovine [23]. BoDV-1 infection may affect on the IFN-tau expression in the embryo. The relatively higher expression of IFN-τ may be essential for ensuring stable pregnancy, and the anti-viral effect of type 1 IFN might contribute to the maintenance of pregnancy in cows infected with BoDV-1. In fact, type 1 IFN has been shown to inhibit BoDV-1 multiplication in an infected animal model [18]. The reports has BoDV-1 down regulated secretion of type 1 IFN through IRF-3 and 7 and Avian borna virus infected cell reduced secretion of type 1 IFN. IFN-τ may be influenced the secretion in fetal side and response in BoDV-1 infected maternal cows [6, 24].

In the present study, we confirmed that the expressions of ISG15 and Mx2 genes were enhanced in cows infected with BoDV-1. Although the levels of type 1 IFN from PBMCs in infected cows have not been investigated, a higher response to IFN-τ and/or increased IFN-τ production during early pregnancy may be important for maintaining pregnancy in cows infected with BoDV-1. Thus, BoDV-1 infection could affect the fecundity and maintenance of pregnancy. Therefore, the conception and maintenance of early pregnancy appears to require a higher response of ISG15 and Mx2 to IFN stimulation in cows infected with BoDV-1. The PBMCs from BoDV-1 infected cows have a clear response to IFN-τ stimulation.

Moreover, BoDV-1 infection may lead to difficulties in conception and maintenance of pregnancy; in these cases, the host may need to produce a greater IFN reaction to overcome this limitation. Repeated insemination and a significant increase in the calving-to-conception interval at BoDV-1 infected cows may relate to change of responsiveness for type 1 IFN. Hence, pregnancy and IFN production should be examined in detail in infected cows in future studies.

## Conclusion

In conclusion, BoDV-1 infection may lead to instability in the maintenance of early pregnancy by interfering with IFN-τ production. BoDV-1 infected dairy cows were lower conception rate than BoDV-1 uninfected cows. The expression of ISG15 and Mx2 during early pregnancy significantly increased in the BoDV-1-infected cows. But in the BoDV-1 uninfected cows did not. Hence, the conception and maintenance of early pregnancy appears to require a higher production of IFN-τ in cows infected with BoDV-1.

## Abbreviations

AI: Artificial insemination
BoDV-1: Borna disease virus-1
IFN: Interferon
IRF: Interferon regulatory factor
ISG: Interferon-stimulated gene
Mx: Myxovirus resistance gene
OAS1: Oligoadenylate synthetase 1
PBMC: Peripheral blood mononuclear cells
STAT: Signal transducers and activator of transcription
TLR: Toll like receptor

## References

[1] Tizard I, Ball J, Stoica G, Payne S (2016). The pathogenesis of bornaviral diseases in mammals. Anim Health Res Rev.

[2] Okamoto M, Furuoka H, Hagiwara K, Kamitani W, Kirisawa R, Ikuta K, Taniyama H (2002). Borna disease in a heifer in Japan. Vet Rec.

[3] Hagiwara K, Kawamoto S, Takahashi H, Nakamura Y, Nakaya T, Hiramune T, Ishihara C, Ikuta K (1997). High prevalence of Borna disease virus infection in healthy sheep in Japan. Clin Diagn Lab Immun.

[4] Bahmani MK, Nowrouzian I, Nakaya T, Nakamura Y, Hagiwara K, Takahashi H, Rad MA, Ikuta K (1996). Varied prevalence of Borna disease virus infection in Arabic, thoroughbred and their cross-bred horses in Iran. Virus Res.

[5] Hagiwara K, Ando T, Koiwa M (2012). The influence of Borna disease viral infection on dairy cow reproduction. J Vet Med Scie.

[6] Meyerholz MM, Mense K, Knaack H, Sandra O, Schmicke M (2016). Pregnancy-Induced ISG-15 and MX-1 Gene Expression is Detected in the Liver of Holstein-Friesian Heifers During Late Peri-Implantation Period. Reproduction in domestic animals. Reprod Domest Anim.

[7] Green JC, Okamura CS, Poock SE, Lucy MC (2010). Measurement of interferon-tau (IFN-tau) stimulated gene expression in blood leukocytes for pregnancy diagnosis within 18-20d after insemination in dairy cattle. Anim Reprod Sci.

[8] Dorniak P, Bazer FW, Spencer TE (2013). Physiology and Endocrinology Symposium: biological role of interferon tau in endometrial function and conceptus elongation. J Anim Sci.

[9] Ruiz-González I, Xu J, Wang X, Burghardt RC, Dunlap KA, Bazer FW (2015). Exosomes, endogenous retroviruses and toll-like receptors: pregnancy recognition in ewes. Reproduction.

[10] Spencer TE, Sandra O, Wolf E (2008). Genes involved in conceptus-endometrial interactions in ruminants: insights from reductionism and thoughts on holistic approaches. Reproduction.

[11] Forde N, Carter F, Spencer TE, Bazer FW, Sandra O, Mansouri-Attia N, Okumu LA, McGettigan PA, Mehta JP, McBride R, O’gaora P, Poche JF, Lonergan P (2011). Conceptus-induced changes in the endometrial transcriptome: how soon does the cow know she is pregnant?. Biol Reprod.

[12] Buraqohain L, Kumar R, Nanda T, Phulia SK, Mohanty AK, Kumar S, Balhara S, Ghuman S, Sinqh I, Balhara AK (2016). Serum MX2 Protein as Candidate Biomarker for Early Pregnancy Diagnosis in Buffalo. Reprod Domest Anim.

[13] Glifford CA, Racicot K, Clark DS, Austin KJ, Hansen TR, Lucy MC, Davies CJ, Ott TL (2007). Regulation of interferon-stimulated genes in peripheral blood leukocytes in pregnant and bred, nonpregnant dairy cows. J Dairy Sci.

[14] Michael DD, Wagner SK, Ocon OM, Talbot NC, Rooke JA, Ealy AD (2006). Granulocyte-macrophage colony-stimulating-factor increases interferon-tau protein secretion in bovine trophectoderm cells. Am J Reprod Immun.

[15] Han H, Austin KJ, Rempel LA, Hansen TR (2006). Low blood ISG15 mRNA and progesterone levels are predictive of non-pregnant dairy cows. J Endocrinol.

[16] Platanias LC (2005). Mechanisms of type-I- and type-II-interferon-mediated signalling. Nat Rev Immunol.

[17] Uddin S, Platanias LC (2004). Mechanisms of type-I interferon signal transduction. J Biochem Mol Biol.

[18] Staeheli P, Sentandreu M, Pagenstecher A, Hausmann J (2001). Alpha/Beta Interferon Promotes Transcription and Inhibits Replication of Borna Disease Virus in Persistently Infected Cells. J Virol.

[19] Puech C, Dedieu L, Chantai I, Rodriguess V (2015). Design and evaluation of unique SYBR Green real-time RT-PCR assay for quantification of five major cytokine in cattle, sheep and goats. BMC Vet Res.

[20] Hagiwara K, Asakawa M, Liao L, Jiang W, Yan S, Chai J, Oku Y, Ikuta K, Ito M (2001). Seroprevalence of Borna disease virus in domestic animals in Xinjiang, China. Vet Microbiol.

[21] Hagiwara K, Okamoto M, Kamitani W, Takamura S, Taniyama H, Tsunoda N, Tanaka H, Iwai H, Ikuta K (2002). Nosological study of Borna disease virus infection in race horses. Vet Microbiol.

[22] Shirozu T, Sasaki K, Kawahara M, Yanagawa Y, Nagano M, Yamauchi N, Takahashi M (2016). Expression dynamics of bovine MX genes in the endometrium and placenta during early to midpregnancy. J Reprod Dev.

[23] Song W, Kao W, Zhai A, Qian J, Li Y, Zhao H, Hu Y, Li H, Zhang F (2013). Borna disease virus nucleoprotein inhibits type I interferon induction through the interferon regulatory factor 7 pathway. Biochem Biophys Res Commun.

[24] Reuter A, Ackermann A, Kothlow S, Rinder M, Kaspers B, Staeheli P (2010). Avian bornaviruses escape recognition by the innate immune system. Viruses.

